# Supplementary material for: Soil elemental changes during human decomposition
Source: PLoS One. 2023 Jun 13;18(6):e0287094. doi: 10.1371/journal.pone.0287094 (PMC10263346; doi:10.1371/journal.pone.0287094)
Supplement: S1 Fig — Treatments (K+ saturated at 25°C, K+ saturated at 300°C, K+ saturated at 550°C, Mg2+ saturated, and Mg2+ with glycol saturated) are shown for soils originating from two separate ARF locations (A and B) having no previous decomposition activity. Clay minerals are identified by d-spacing in nm. (DOCX) [file pone.0287094.s001.docx]

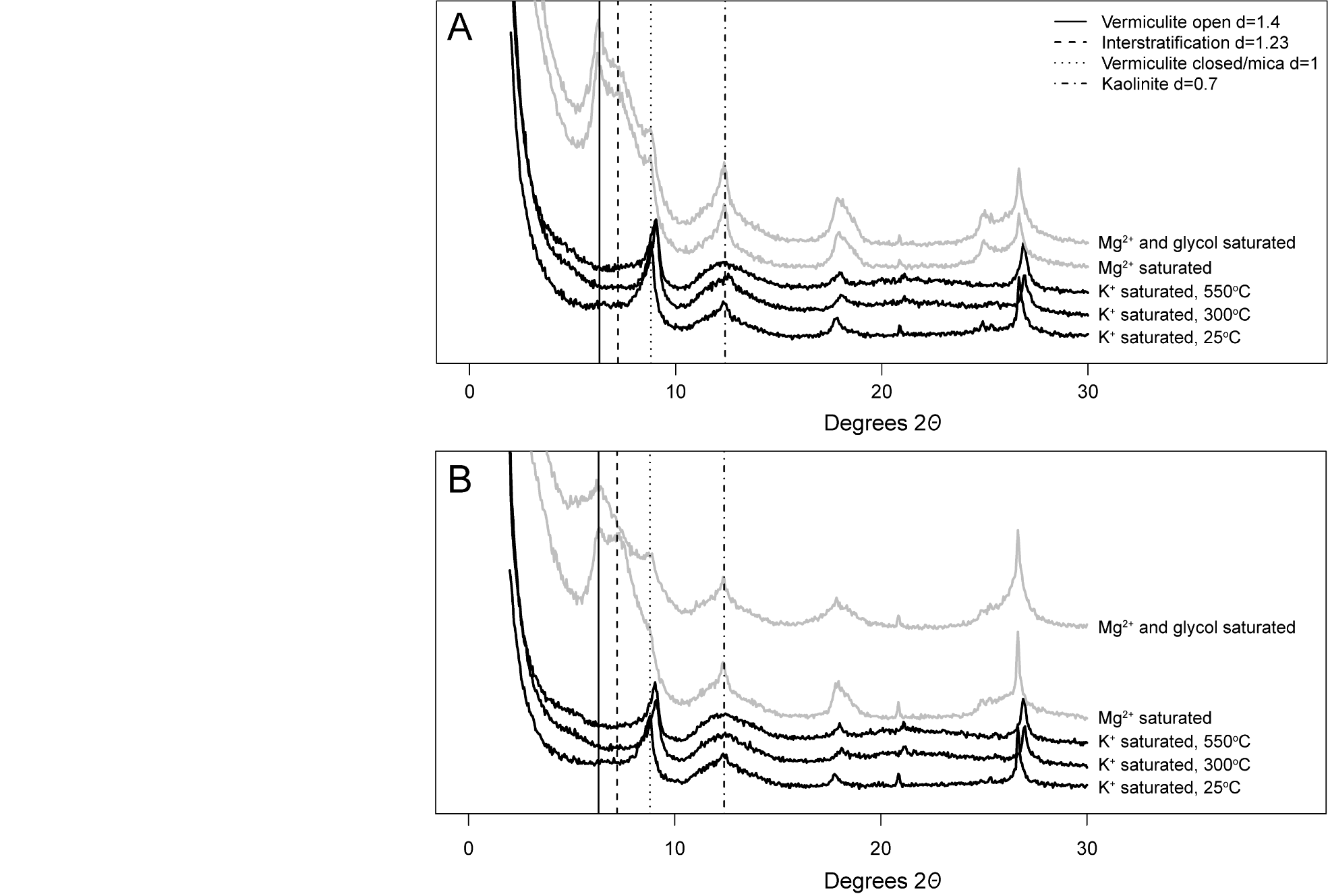


**Supplementary Figure S2: X-ray diffractograms of clay minerals.** Treatments (K^+^ saturated at 25 ^o^C, K^+^ saturated at 300 ^o^C, K^+^ saturated at 550 ^o^C, Mg^2+^ saturated, and Mg^2+^ with glycol saturated) are shown for soils originating from two separate ARF locations (A and B) having no previous decomposition activity. Clay minerals are identified by d-spacing in nm.
