# Supplementary material for: Soil elemental changes during human decomposition
Source: PLoS One. 2023 Jun 13;18(6):e0287094. doi: 10.1371/journal.pone.0287094 (PMC10263346; doi:10.1371/journal.pone.0287094)
Supplement: S1 Table — Selected elements are those that occur in greatest abundance in the human body and are listed in approximate order of percentage found in the human body from greatest to least. Data are means ± standard deviations for n = 3 replicate donors. Impacted soils that significantly differ from controls based upon Welch T-tests (p < 0.05) are presented in bold type. Asterisks indicate levels of significance: * p < 0.05, ** p < 0.01, ***p < 0.001. (DOCX) [file pone.0287094.s002.docx]

| **Table S1: Elemental concentrations in soil during human decomposition.** Selected elements are those that occur in greatest abundance in the human body and are listed in approximate order of percentage found in the human body from greatest to least**.** Data are means ± standard deviations for n = 3 replicate donors. Impacted soils that significantly differ from controls based upon Welch T-tests (p < 0.05) are presented in bold type. Asterisks indicate levels of significance: * p < 0.05, ** p < 0.01, ***p < 0.001. | | | | | | | | | | |
| --- | --- | --- | --- | --- | --- | --- | --- | --- | --- | --- |
| **Study day** | **Location** | **pH** | **EC** | **Ca**  **(µg gdw^-1^)** | **P**  **(µg gdw^-1^)** | **K**  **(ug gdw^-1^)** | **S**  **(µg gdw^-1^)** | **Na**  **(µg gdw^-1^)** | **Mg**  **(µg gdw^-1^)** |  |
| **0** | **decomposition** | 7.1 ± 0.4 | 46.8 ± 7.4 | 90.7 ± 62 | 1.1 ± 1.1 | 22.6 ± 16.3 | 7 ± 3.5 | 2.6 ± 0.8 | 7.1 ± 4.4 |  |
|  | **control** | 7.6 ± 0.1 | 75.9 ± 25.5 | 156.1 ± 52.6 | 0.9 ± 0.7 | 30.8 ± 23.4 | 6.5 ± 2.1 | 1.9 ± 0.9 | 8.9 ± 4.4 |  |
| **3** | **decomposition** | 7.3 ± 0.3 | 49.1 ± 23.7 | 119.7 ± 73.3 | 1 ± 1.1 | 24.1 ± 21.5 | 6.9 ± 3.3 | 4.4 ± 1.3 | 8.9 ± 4.6 |  |
|  | **control** | 7.5 ± 0.3 | 70 ± 36.9 | 150.2 ± 34.6 | 1 ± 0.5 | 28 ± 22 | 6.3 ± 1.3 | 2 ± 0.5 | 8.6 ± 2.9 |  |
| **5** | **decomposition** | 7.2 ± 0.2 | 66.3 ± 53.3 | 97.5 ± 71.4 | 1.2 ± 0.9 | 26.2 ± 16.1 | 6.6 ± 2.5 | 8.3 ± 4.7 | 7.6 ± 4.6 |  |
|  | **control** | 7.5 ± 0 | 61 ± 25.9 | 131.4 ± 42.5 | 0.8 ± 0.5 | 23.3 ± 20.7 | 5.3 ± 1.5 | 1.5 ± 0.2 | 7.6 ± 3.3 |  |
| **7** | **decomposition** | 7.2 ± 0.4 | 54.5 ± 31.5 | 98.9 ± 69.3 | 0.9 ± 1 | 23 ± 19.7 | 6.8 ± 3.7 | 6.3 ± 3.5 | 7.7 ± 5 |  |
|  | **control** | 7.7 ± 0.2 | 87.8 ± 37.5 | 135.9 ± 98.4 | 0.8 ± 0.4 | 15 ± 3.3 | 6.9 ± 0.9 | 3.1 ± 1.5 | 6.8 ± 1.8 |  |
| **10** | **decomposition** | 6.9 ± 0.3 | 119.1 ± 124.8 | 122.7 ± 87.8 | 0.6 ± 0.3 | 25 ± 21.9 | 7.5 ± 4.5 | 18.6 ± 21.9 | 9.1 ± 4.9 |  |
|  | **control** | 7.5 ± 0.2 | 83.6 ± 47.2 | 157.6 ± 61.8 | 1.1 ± 0.7 | 26.9 ± 19.6 | 6.5 ± 2.9 | 1.7 ± 0.3 | 8.9 ± 3.6 |  |
| **14** | **decomposition** | 7 ± 0.3 | 135.6 ± 103.8 | 119.4 ± 72.8 | 1.2 ± 1.2 | 29.5 ± 26.2 | 10.2 ± 5.8 | 23.7 ± 14.4 | 8.7 ± 4 |  |
|  | **control** | 7.4 ± 0.3 | 81.1 ± 44.6 | 166.6 ± 79.3 | 1 ± 0.8 | 26.6 ± 21.1 | 6.4 ± 2.6 | 1.6 ± 0.1 | 8.1 ± 3.2 |  |
| **17** | **decomposition** | 7 ± 0.6 | 208.5 ± 127.7 | 194.5 ± 95.7 | 12.3 ± 11.4 | 72.6 ± 45.4 | 22.5 ± 12.8 | 102.2 ± 74 | 15.9 ± 5.9 |  |
|  | **control** | 7.5 ± 0.2 | 92.9 ± 46.7 | 154.3 ± 36 | 1 ± 0.7 | 36.7 ± 25.9 | 6.8 ± 1.9 | 1.8 ± 0.4 | 8.7 ± 3 |  |
| **19** | **decomposition** | 7 ± 0.4 | 297.8 ± 201.9 | 252.5 ± 136.8 | 4.6 ± 4.7 | 62.5 ± 34.3 | 22.8 ± 10.3 | 108 ± 68.6 | 19.9 ± 10.7 |  |
|  | **control** | 7.1 ± 0.3 | 69.5 ± 10 | 114.6 ± 21.3 | 0.9 ± 0.6 | 31.3 ± 22.8 | 6.5 ± 1.9 | 2.3 ± 0.5 | 7.1 ± 3.6 |  |
| **21** | **decomposition** | 6.8 ± 0.4 | 278.1 ± 178.6 | 212.8 ± 152 | 3.7 ± 4.6 | 52.4 ± 40.8 | 19.9 ± 8.6 | 138.5 ± 161.7 | 16.6 ± 9.8 |  |
|  | **control** | 7.3 ± 0 | 107 ± 78.3 | 108.4 ± 40.3 | 0.8 ± 0.5 | 20.7 ± 15.8 | 4.8 ± 1.3 | 2.1 ± 0.9 | 6.4 ± 3.8 |  |
| **28** | **decomposition** | 6.4 ± 0.4 | 293.2 ± 102.4 | 297.1 ± 186.9 | 12.8 ± 18.4 | 92 ± 76.5 | 24.7 ± 7.6 | 111.4 ± 76.5 | 26.4 ± 17.1 |  |
|  | **control** | 7.3 ± 0.1 | 67.5 ± 36 | 126.3 ± 38.2 | 1 ± 0.8 | 30.6 ± 16.2 | 6.3 ± 1.3 | 2.1 ± 0.5 | 7.1 ± 3.5 |  |
| **33** | **decomposition** | **6 ± 0.5*** | **355.9 ± 38.3**** | 331.8 ± 101.8 | 19.8 ± 20 | 121 ± 63.4 | **33.3 ± 11.2*** | 188.9 ± 121.8 | **31 ± 8.2*** |  |
|  | **control** | 7.5 ± 0.2 | 89.2 ± 43.9 | 156.4 ± 50.6 | 0.9 ± 0.5 | 26.1 ± 18 | 5.7 ± 1.9 | 1.9 ± 0.4 | 8 ± 2.9 |  |
| **35** | **decomposition** | 6.3 ± 0.5 | **266.7 ± 29.8**** | 211.8 ± 73.5 | **4.1 ± 0.4**** | 76.5 ± 17.3 | **26.6 ± 3.9**** | **131.5 ± 33.5*** | 19.1 ± 8.9 |  |
|  | **control** | 7.2 ± 0.2 | 86.4 ± 37.1 | 145.8 ± 54.9 | 0.9 ± 0.6 | 29.4 ± 26.7 | 8.7 ± 4.4 | 2.4 ± 0.6 | 7.7 ± 3.3 |  |
| **38** | **decomposition** | 6 ± 0.6 | **400.4 ± 84.9*** | 302.2 ± 244.7 | 20.7 ± 17 | 118.3 ± 50.1 | **33 ± 1.4***** | **213.2 ± 82.3*** | 27.7 ± 18.3 |  |
|  | **control** | 6.9 ± 0.1 | 54.4 ± 9.7 | 128 ± 56.5 | 1 ± 0.4 | 29.5 ± 22 | 6 ± 1.5 | 2.5 ± 1.2 | 7.6 ± 5 |  |
| **40** | **decomposition** | **5.9 ± 0.4*** | **240.9 ± 49.5*** | 240.2 ± 103.4 | 2.9 ± 2.7 | 66.3 ± 27.3 | 17.8 ± 7.7 | 128.4 ± 75.8 | 21.3 ± 13.5 |  |
|  | **control** | 6.8 ± 0.1 | 52.4 ± 14.2 | 124.1 ± 56 | 1.1 ± 0.7 | 27.1 ± 19.9 | 6.4 ± 2.4 | 2.2 ± 0.9 | 7.5 ± 3.9 |  |
| **42** | **decomposition** | **6 ± 0.4*** | **346.4 ± 38**** | 307.4 ± 97.7 | 12.8 ± 7.9 | **105.7 ± 19.4*** | 30 ± 12.2 | 145.1 ± 67.9 | **28.7 ± 9.6*** |  |
|  | **control** | 7.2 ± 0.2 | 59.5 ± 5 | 136.4 ± 41.1 | 1.1 ± 0.6 | 30.9 ± 23 | 7 ± 1.8 | 2.4 ± 0.4 | 7.6 ± 3.9 |  |
| **45** | **decomposition** | **5.8 ± 0.3**** | **321.8 ± 68.6*** | 272.3 ± 71.5 | **15.7 ± 4.5*** | **120.4 ± 32*** | 40.4 ± 17.9 | 190.6 ± 103.7 | **27.4 ± 4.5**** |  |
|  | **control** | 7.2 ± 0.2 | 43.5 ± 1.9 | 129 ± 9.9 | 1.2 ± 0.6 | 28.6 ± 18 | 8.4 ± 2 | 3.2 ± 2.1 | 7.4 ± 2.7 |  |
| **47** | **decomposition** | **5.9 ± 0.2**** | **366 ± 103.2*** | **320.8 ± 82.5*** | 10.1 ± 5.7 | **86.4 ± 14*** | **23.9 ± 6.5*** | **140.3 ± 41.5*** | **28.1 ± 3.5**** |  |
|  | **control** | 7.2 ± 0 | 39.5 ± 18.6 | 120.2 ± 52.5 | 1.1 ± 0.8 | 24 ± 21.6 | 6 ± 2.2 | 2 ± 0.5 | 6.8 ± 4.4 |  |
| **49** | **decomposition** | **5.8 ± 0.1***** | **434.7 ± 103.5*** | 400.5 ± 126.3 | 19.4 ± 10.1 | **119.7 ± 29.2*** | **31 ± 7.7*** | **174.5 ± 67*** | **36.1 ± 1.4**** |  |
|  | **control** | 7.2 ± 0.1 | 66.1 ± 9.4 | 128.8 ± 37.9 | 1.2 ± 0.7 | 25.5 ± 22.9 | 8 ± 2.6 | 3.3 ± 1.3 | 7.6 ± 5.1 |  |
| **54** | **decomposition** | **6 ± 0.1**** | 317.2 ± 136.7 | 246.5 ± 163.5 | 6.5 ± 3.3 | **97.8 ± 21.8*** | **28 ± 6.4*** | 157.5 ± 88.9 | 21.3 ± 8 |  |
|  | **control** | 7.2 ± 0.2 | 62.7 ± 5.3 | 139.1 ± 44.4 | 0.9 ± 0.6 | 29.4 ± 18.9 | 6.4 ± 2.1 | 2.3 ± 0.4 | 7.2 ± 2.2 |  |
| **56** | **decomposition** | **6.1 ± 0.6*** | **359.3 ± 32***** | 164.9 ± 80.6 | 18.9 ± 12.5 | **121.7 ± 17.2*** | **37.5 ± 2.8***** | 197.9 ± 92.7 | 18.2 ± 9.1 |  |
|  | **control** | 7.6 ± 0.2 | 75.5 ± 34.7 | 133.1 ± 33.1 | 0.9 ± 0.7 | 32.6 ± 33 | 6.9 ± 2 | 2.5 ± 0.3 | 7.4 ± 3.8 |  |
| **61** | **decomposition** | **6.2 ± 0.1***** | 297.3 ± 156.9 | 189.1 ± 87.4 | 19.4 ± 15 | 119.1 ± 45.7 | 30.1 ± 13.9 | 169.7 ± 89 | **20.6 ± 3.2**** |  |
|  | **control** | 7.6 ± 0.1 | 66.5 ± 21.1 | 137.5 ± 26.6 | 1 ± 0.4 | 32.3 ± 23.9 | 6.8 ± 1.3 | 2.3 ± 0.6 | 7.3 ± 2.7 |  |
| **66** | **decomposition** | **6.4 ± 0.4*** | **259.4 ± 57.4*** | 185.2 ± 69.7 | 18 ± 14.8 | 96.7 ± 36.5 | 26.3 ± 10.8 | 135.4 ± 64.5 | **19.5 ± 4.7*** |  |
|  | **control** | 7.4 ± 0.1 | 57 ± 32.1 | 122.1 ± 38.1 | 1.1 ± 0.9 | 33.8 ± 30.7 | 6.4 ± 0.8 | 2.7 ± 1.3 | 6.8 ± 2.4 |  |
| **75** | **decomposition** | **6.5 ± 0.4*** | **257.8 ± 38.1*** | **209.6 ± 21.6**** | 4.3 ± 3.5 | 75.8 ± 35.6 | 24.4 ± 12.5 | 152.6 ± 99.1 | **20.2 ± 4.2*** |  |
|  | **control** | 7.4 ± 0.1 | 45.6 ± 4.5 | 104.2 ± 18.9 | 0.8 ± 0.6 | 27.7 ± 25.3 | 5.8 ± 1.9 | 2.4 ± 1.1 | 6.1 ± 2.9 |  |
| **89** | **decomposition** | **6 ± 0.2**** | 387.9 ± 158 | 179.4 ± 114.4 | 12 ± 10.7 | 92.2 ± 35.2 | 24.9 ± 14.1 | 187 ± 112.3 | 17.9 ± 7 |  |
|  | **control** | 7.4 ± 0.2 | 92.9 ± 41.4 | 156.4 ± 45.9 | 1 ± 0.3 | 30.7 ± 12.8 | 5.9 ± 1.2 | 2.2 ± 0.4 | 7.7 ± 2.1 |  |
| **103** | **decomposition** | **6.2 ± 0.3*** | 318.4 ± 118.6 | 175.4 ± 133.1 | 15.3 ± 17.5 | 113.6 ± 85.4 | 31.8 ± 21.9 | 184.6 ± 126.3 | 20 ± 10.5 |  |
|  | **control** | 7.5 ± 0 | 85.4 ± 55.5 | 171.3 ± 67.5 | 0.9 ± 0.5 | 26.7 ± 14.7 | 6.8 ± 2.2 | 2.5 ± 0.7 | 8.3 ± 2.5 |  |
| **117** | **decomposition** | 6.3 ± 0.5 | 455.9 ± 201 | 175.9 ± 92.1 | 19.6 ± 20.2 | 110.5 ± 56.5 | 33.5 ± 19.7 | 232.7 ± 161.6 | 18.6 ± 6.4 |  |
|  | **control** | 7.3 ± 0.2 | 89.7 ± 37.7 | 144.6 ± 39.2 | 0.7 ± 0.4 | 17.6 ± 12.5 | 6.1 ± 1.8 | 2.2 ± 0.4 | 6.9 ± 2.6 |  |
| **122** | **decomposition** | **6.1 ± 0.3*** | 266.1 ± 180.7 | 154.6 ± 63.7 | 6.6 ± 5.9 | 87.3 ± 42.5 | 23.3 ± 7.8 | 168.1 ± 77.3 | 16 ± 4.3 |  |
|  | **control** | 7.3 ± 0.2 | 49.8 ± 14.4 | 144.2 ± 36.3 | 0.8 ± 0.6 | 23.4 ± 13.7 | 5.9 ± 0.7 | 2.4 ± 0.8 | 7.4 ± 2.9 |  |
