# Supplementary material for: Soil elemental changes during human decomposition
Source: PLoS One. 2023 Jun 13;18(6):e0287094. doi: 10.1371/journal.pone.0287094 (PMC10263346; doi:10.1371/journal.pone.0287094)
Supplement: S2 Table — Welch T-tests assuming unequal sample variances were conducted at each sampling time point in order to compare differences between impacted soils and controls for pH, electrical conductivity (EC), calcium (Ca), phosphorus (P), potassium (K), sulfur (S), sodium (Na), magnesium (Mg), Iron (Fe), copper (Cu), manganese (Mn), zinc (Zn), selenium (Se), cobalt (Co), boron, (B), and aluminum (Al). P values are shown, and significant differences based upon p < 0.05 are presented in bold type. Asterisks indicate levels of significance: * p < 0.05, ** p < 0.01, ***p < 0.001. (DOCX) [file pone.0287094.s003.docx]

| **Table S2. Results of Welch T-tests between decomposition-impacted soils and controls.** Welch T-tests assuming unequal sample variances were conducted at each sampling time point in order to compare differences between impacted soils and controls for pH, electrical conductivity (EC), calcium (Ca), phosphorus (P), potassium (K), sulfur (S), sodium (Na), magnesium (Mg), Iron (Fe), copper (Cu), manganese (Mn), zinc (Zn), selenium (Se), cobalt (Co), boron, (B), and aluminum (Al). P values are shown, and significant differences based upon p < 0.05 are presented in bold type. Asterisks indicate levels of significance: * p < 0.05, ** p < 0.01, ***p < 0.001. | | | | | | | | | | | | | | | | |
| --- | --- | --- | --- | --- | --- | --- | --- | --- | --- | --- | --- | --- | --- | --- | --- | --- |
| **Study Day** | **pH** | **EC** | **Ca** | **P** | **K** | **S** | **Na** | **Mg** | **Fe** | **Cu** | **Mn** | **Zn** | **Se** | **Co** | **B** | **Al** |
| **0** | 0.188 | 0.179 | 0.238 | 0.854 | 0.650 | 0.846 | 0.332 | 0.648 | 0.713 | 0.399 | 0.573 | 0.452 | 0.532 | 0.838 | 0.657 | 0.619 |
| **3** | 0.491 | 0.463 | 0.564 | 0.928 | 0.837 | 0.815 | 0.072 | 0.935 | 0.960 | 0.274 | 0.854 | 0.994 | 0.558 | 0.873 | 0.549 | 0.935 |
| **5** | 0.092 | 0.887 | 0.528 | 0.639 | 0.858 | 0.472 | 0.130 | 0.989 | 0.661 | 0.578 | 0.873 | 0.357 | 0.251 | 0.753 | 0.701 | 0.994 |
| **7** | 0.185 | 0.306 | 0.626 | 0.865 | 0.557 | 0.964 | 0.260 | 0.792 | 0.811 | 0.218 | 0.908 | 0.763 | 0.625 | 0.745 | 0.979 | 0.841 |
| **10** | 0.061 | 0.681 | 0.607 | 0.355 | 0.917 | 0.759 | 0.315 | 0.955 | 0.991 | 0.456 | 0.710 | 0.958 | 0.676 | 0.730 | 0.757 | 0.660 |
| **14** | 0.198 | 0.471 | 0.490 | 0.809 | 0.889 | 0.385 | 0.116 | 0.847 | 0.801 | 0.472 | 0.411 | 0.807 | 0.838 | 0.360 | 0.972 | 0.938 |
| **17** | 0.259 | 0.253 | 0.552 | 0.230 | 0.316 | 0.166 | 0.143 | 0.157 | 0.821 | 0.582 | **0.005**** | 0.542 | 0.394 | 0.445 | 0.657 | 0.587 |
| **19** | 0.638 | 0.189 | 0.221 | 0.309 | 0.270 | 0.106 | 0.117 | 0.165 | 0.674 | 0.659 | 0.353 | 0.420 | 0.085 | 0.512 | 0.696 | 0.172 |
| **21** | 0.189 | 0.234 | 0.356 | 0.385 | 0.311 | 0.091 | 0.281 | 0.204 | 0.989 | 0.762 | 0.296 | **0.014*** | 0.310 | 0.236 | 0.637 | 0.194 |
| **28** | 0.066 | 0.050 | 0.252 | 0.382 | 0.297 | 0.050 | 0.132 | 0.186 | 0.075 | 0.801 | 0.228 | 0.071 | 0.105 | 0.595 | 0.529 | **0.004**** |
| **33** | **0.018*** | **0.001**** | 0.077 | 0.243 | 0.113 | **0.047*** | 0.117 | **0.029*** | 0.904 | 0.314 | 0.122 | 0.167 | 0.088 | 0.061 | 0.369 | 0.086 |
| **35** | 0.051 | **0.003**** | 0.286 | **0.003**** | 0.073 | **0.006**** | **0.022*** | 0.145 | 0.843 | 0.504 | 0.376 | 0.238 | 0.148 | 0.267 | 0.423 | 0.054 |
| **38** | 0.118 | **0.018*** | 0.343 | 0.182 | 0.075 | **<0.001***** | **0.047*** | 0.193 | 0.182 | **0.037*** | 0.231 | 0.124 | 0.148 | 0.264 | 0.395 | 0.280 |
| **40** | **0.035*** | **0.016*** | 0.183 | 0.368 | 0.122 | 0.113 | 0.102 | 0.213 | 0.909 | 0.600 | 0.373 | 0.052 | 0.141 | 0.247 | 0.440 | 0.171 |
| **42** | **0.025*** | **0.005**** | 0.077 | 0.125 | **0.013*** | 0.078 | 0.068 | **0.048*** | 0.364 | 0.497 | 0.326 | 0.144 | 0.108 | 0.276 | 0.453 | 0.093 |
| **45** | **0.003**** | **0.020*** | 0.071 | **0.030*** | **0.021*** | 0.088 | 0.089 | **0.005**** | 0.116 | 0.417 | 0.235 | **0.026*** | **0.048*** | 0.177 | 0.392 | 0.123 |
| **47** | **0.007**** | **0.028*** | **0.031*** | 0.109 | **0.019*** | **0.030*** | **0.029*** | **0.003**** | 0.167 | 0.464 | 0.306 | 0.117 | 0.312 | 0.325 | 0.373 | 0.458 |
| **49** | **<0.001***** | **0.025*** | 0.055 | 0.089 | **0.013*** | **0.026*** | **0.047*** | **0.007**** | 0.311 | 0.249 | 0.133 | **0.047*** | 0.075 | 0.182 | 0.386 | 0.101 |
| **54** | **0.002**** | 0.084 | 0.374 | 0.093 | **0.015*** | **0.020*** | 0.094 | 0.083 | 0.454 | 0.111 | 0.126 | **0.029*** | 0.139 | 0.161 | 0.461 | 0.058 |
| **56** | **0.043*** | **<0.001***** | 0.577 | 0.130 | **0.025*** | **<0.001***** | 0.067 | 0.163 | **0.007**** | 0.079 | 0.346 | 0.060 | 0.191 | 0.259 | 0.466 | 0.457 |
| **61** | **<0.001***** | 0.123 | 0.417 | 0.167 | 0.061 | 0.099 | 0.083 | **0.006**** | **0.037*** | 0.086 | 0.096 | **0.047*** | **0.013*** | 0.135 | 0.532 | 0.752 |
| **66** | **0.029*** | **0.012*** | 0.260 | 0.185 | 0.086 | 0.085 | 0.071 | **0.025*** | 0.953 | 0.337 | 0.139 | 0.146 | **0.020*** | 0.094 | 0.462 | 0.094 |
| **75** | **0.046*** | **0.010*** | **0.003**** | 0.225 | 0.136 | 0.121 | 0.120 | **0.012*** | 0.610 | 0.316 | 0.334 | 0.223 | 0.198 | 0.228 | 0.457 | 0.996 |
| **89** | **0.001**** | 0.075 | 0.770 | 0.216 | 0.080 | 0.143 | 0.104 | 0.121 | **0.005**** | 0.111 | **0.005**** | 0.059 | **0.009**** | **0.042*** | 0.369 | 0.080 |
| **103** | **0.020*** | 0.058 | 0.965 | 0.292 | 0.217 | 0.185 | 0.130 | 0.190 | **0.008**** | 0.213 | 0.066 | 0.193 | 0.241 | 0.193 | 0.553 | **0.025*** |
| **117** | 0.062 | 0.083 | 0.630 | 0.246 | 0.098 | 0.137 | 0.132 | 0.070 | 0.128 | **0.007**** | 0.131 | 0.061 | **<0.001***** | 0.160 | 0.621 | 0.151 |
| **122** | **0.011*** | 0.173 | 0.821 | 0.232 | 0.110 | 0.061 | 0.066 | 0.052 | 0.180 | 0.112 | 0.204 | 0.091 | 0.060 | 0.329 | 0.266 | 0.054 |
