# Supplementary material for: Soil elemental changes during human decomposition
Source: PLoS One. 2023 Jun 13;18(6):e0287094. doi: 10.1371/journal.pone.0287094 (PMC10263346; doi:10.1371/journal.pone.0287094)
Supplement: S3 Table — Charge concentrations (μmolc gdw-1) of exchangeable cations (Na+, K+, Ca2+, Mg2+) in controls and decomposition-impacted soils are shown for the entire study. Data are means ± standard deviations for n = 3 replicate donors. (DOCX) [file pone.0287094.s004.docx]

| **Table S3: Charge concentrations of exchangeable cations in soil during human decomposition.** Charge concentrations (µmol_c_ gdw^-1^) of exchangeable cations (Na^+^, K^+^, Ca^2+^, Mg^2+^) in controls and decomposition-impacted soils are shown for the entire study. Data are means ± standard deviations for n = 3 replicate donors. | | | | | | |
| --- | --- | --- | --- | --- | --- | --- |
| **Study day** | **Location** | **Na^+^**  **(µmol_c_ gdw^-1^)** | **K^+^**  **(µmol_c_ gdw^-1^)** | **Ca^2+^**  **(µmol_c_ gdw^-1^)** | **Mg^2+^**  **(µmol_c_ gdw^-1^)** |  |
| **0** | **decomposition** | 0.1 ± 0 | 0.6 ± 0.4 | 4.5 ± 3.1 | 0.6 ± 0.4 |  |
|  | **control** | 0.1 ± 0 | 0.8 ± 0.6 | 7.8 ± 2.6 | 0.7 ± 0.4 |  |
| **3** | **decomposition** | 0.2 ± 0.1 | 0.6 ± 0.6 | 6 ± 3.7 | 0.7 ± 0.4 |  |
|  | **control** | 0.1 ± 0 | 0.7 ± 0.6 | 7.5 ± 1.7 | 0.7 ± 0.2 |  |
| **5** | **decomposition** | 0.4 ± 0.2 | 0.7 ± 0.4 | 4.9 ± 3.6 | 0.6 ± 0.4 |  |
|  | **control** | 0.1 ± 0 | 0.6 ± 0.5 | 6.6 ± 2.1 | 0.6 ± 0.3 |  |
| **7** | **decomposition** | 0.3 ± 0.2 | 0.6 ± 0.5 | 4.9 ± 3.5 | 0.6 ± 0.4 |  |
|  | **control** | 0.1 ± 0.1 | 0.4 ± 0.1 | 6.8 ± 4.9 | 0.6 ± 0.1 |  |
| **10** | **decomposition** | 0.8 ± 1 | 0.6 ± 0.6 | 6.1 ± 4.4 | 0.8 ± 0.4 |  |
|  | **control** | 0.1 ± 0 | 0.7 ± 0.5 | 7.9 ± 3.1 | 0.7 ± 0.3 |  |
| **14** | **decomposition** | 1 ± 0.6 | 0.8 ± 0.7 | 6 ± 3.6 | 0.7 ± 0.3 |  |
|  | **control** | 0.1 ± 0 | 0.7 ± 0.5 | 8.3 ± 4 | 0.7 ± 0.3 |  |
| **17** | **decomposition** | 4.4 ± 3.2 | 1.9 ± 1.2 | 9.7 ± 4.8 | 1.3 ± 0.5 |  |
|  | **control** | 0.1 ± 0 | 0.9 ± 0.7 | 7.7 ± 1.8 | 0.7 ± 0.2 |  |
| **19** | **decomposition** | 4.7 ± 3 | 1.6 ± 0.9 | 12.6 ± 6.8 | 1.6 ± 0.9 |  |
|  | **control** | 0.1 ± 0 | 0.8 ± 0.6 | 5.7 ± 1.1 | 0.6 ± 0.3 |  |
| **21** | **decomposition** | 6 ± 7 | 1.3 ± 1 | 10.6 ± 7.6 | 1.4 ± 0.8 |  |
|  | **control** | 0.1 ± 0 | 0.5 ± 0.4 | 5.4 ± 2 | 0.5 ± 0.3 |  |
| **28** | **decomposition** | 4.8 ± 3.3 | 2.4 ± 2 | 14.8 ± 9.3 | 2.2 ± 1.4 |  |
|  | **control** | 0.1 ± 0 | 0.8 ± 0.4 | 6.3 ± 1.9 | 0.6 ± 0.3 |  |
| **33** | **decomposition** | 8.2 ± 5.3 | 3.1 ± 1.6 | 16.6 ± 5.1 | 2.6 ± 0.7 |  |
|  | **control** | 0.1 ± 0 | 0.7 ± 0.5 | 7.8 ± 2.5 | 0.7 ± 0.2 |  |
| **35** | **decomposition** | 5.7 ± 1.5 | 2 ± 0.4 | 10.6 ± 3.7 | 1.6 ± 0.7 |  |
|  | **control** | 0.1 ± 0 | 0.8 ± 0.7 | 7.3 ± 2.7 | 0.6 ± 0.3 |  |
| **38** | **decomposition** | 9.3 ± 3.6 | 3 ± 1.3 | 15.1 ± 12.2 | 2.3 ± 1.5 |  |
|  | **control** | 0.1 ± 0.1 | 0.8 ± 0.6 | 6.4 ± 2.8 | 0.6 ± 0.4 |  |
| **40** | **decomposition** | 5.6 ± 3.3 | 1.7 ± 0.7 | 12 ± 5.2 | 1.8 ± 1.1 |  |
|  | **control** | 0.1 ± 0 | 0.7 ± 0.5 | 6.2 ± 2.8 | 0.6 ± 0.3 |  |
| **42** | **decomposition** | 6.3 ± 3 | 2.7 ± 0.5 | 15.3 ± 4.9 | 2.4 ± 0.8 |  |
|  | **control** | 0.1 ± 0 | 0.8 ± 0.6 | 6.8 ± 2.1 | 0.6 ± 0.3 |  |
| **45** | **decomposition** | 8.3 ± 4.5 | 3.1 ± 0.8 | 13.6 ± 3.6 | 2.3 ± 0.4 |  |
|  | **control** | 0.1 ± 0.1 | 0.7 ± 0.5 | 6.4 ± 0.5 | 0.6 ± 0.2 |  |
| **47** | **decomposition** | 6.1 ± 1.8 | 2.2 ± 0.4 | 16 ± 4.1 | 2.3 ± 0.3 |  |
|  | **control** | 0.1 ± 0 | 0.6 ± 0.6 | 6 ± 2.6 | 0.6 ± 0.4 |  |
| **49** | **decomposition** | 7.6 ± 2.9 | 3.1 ± 0.7 | 20 ± 6.3 | 3 ± 0.1 |  |
|  | **control** | 0.1 ± 0.1 | 0.7 ± 0.6 | 6.4 ± 1.9 | 0.6 ± 0.4 |  |
| **54** | **decomposition** | 6.9 ± 3.9 | 2.5 ± 0.6 | 12.3 ± 8.2 | 1.8 ± 0.7 |  |
|  | **control** | 0.1 ± 0 | 0.8 ± 0.5 | 6.9 ± 2.2 | 0.6 ± 0.2 |  |
| **56** | **decomposition** | 8.6 ± 4 | 3.1 ± 0.4 | 8.2 ± 4 | 1.5 ± 0.7 |  |
|  | **control** | 0.1 ± 0 | 0.8 ± 0.8 | 6.6 ± 1.7 | 0.6 ± 0.3 |  |
| **61** | **decomposition** | 7.4 ± 3.9 | 3 ± 1.2 | 9.4 ± 4.4 | 1.7 ± 0.3 |  |
|  | **control** | 0.1 ± 0 | 0.8 ± 0.6 | 6.9 ± 1.3 | 0.6 ± 0.2 |  |
| **66** | **decomposition** | 5.9 ± 2.8 | 2.5 ± 0.9 | 9.2 ± 3.5 | 1.6 ± 0.4 |  |
|  | **control** | 0.1 ± 0.1 | 0.9 ± 0.8 | 6.1 ± 1.9 | 0.6 ± 0.2 |  |
| **75** | **decomposition** | 6.6 ± 4.3 | 1.9 ± 0.9 | 10.5 ± 1.1 | 1.7 ± 0.3 |  |
|  | **control** | 0.1 ± 0 | 0.7 ± 0.6 | 5.2 ± 0.9 | 0.5 ± 0.2 |  |
| **89** | **decomposition** | 8.1 ± 4.9 | 2.4 ± 0.9 | 9 ± 5.7 | 1.5 ± 0.6 |  |
|  | **control** | 0.1 ± 0 | 0.8 ± 0.3 | 7.8 ± 2.3 | 0.6 ± 0.2 |  |
| **103** | **decomposition** | 8 ± 5.5 | 2.9 ± 2.2 | 8.8 ± 6.6 | 1.6 ± 0.9 |  |
|  | **control** | 0.1 ± 0 | 0.7 ± 0.4 | 8.5 ± 3.4 | 0.7 ± 0.2 |  |
| **117** | **decomposition** | 10.1 ± 7 | 2.8 ± 1.4 | 8.8 ± 4.6 | 1.5 ± 0.5 |  |
|  | **control** | 0.1 ± 0 | 0.5 ± 0.3 | 7.2 ± 2 | 0.6 ± 0.2 |  |
| **122** | **decomposition** | 7.3 ± 3.4 | 2.2 ± 1.1 | 7.7 ± 3.2 | 1.3 ± 0.4 |  |
|  | **control** | 0.1 ± 0 | 0.6 ± 0.4 | 7.2 ± 1.8 | 0.6 ± 0.2 |  |
