# Supplementary material for: Soil elemental changes during human decomposition
Source: PLoS One. 2023 Jun 13;18(6):e0287094. doi: 10.1371/journal.pone.0287094 (PMC10263346; doi:10.1371/journal.pone.0287094)
Supplement: S4 Table — With the exception of Aluminum (Al), selected elements are those that occur in trace abundance in the human body and are listed in approximate order of percentage found in the human body from greatest to least. Aluminosilicates are common to East Tennessee soils, and Al is included in order to demonstrate increased mobility in soil under decreased pH found in decomposition. Data are means ± standard deviations for n = 3 replicate donors. Impacted soils that significantly differ from controls based upon Welch T-tests (p < 0.05) are presented in bold type. Asterisks indicate levels of significance: * p < 0.05, ** p < 0.01, ***p < 0.001. (DOCX) [file pone.0287094.s005.docx]

| **Table S4: Trace elemental concentrations in soil during human decomposition.** With the exception of Aluminum (Al), selected elements are those that occur in trace abundance in the human body and are listed in approximate order of percentage found in the human body from greatest to least. Aluminosilicates are common to East Tennessee soils, and Al is included in order to demonstrate increased mobility in soil under decreased pH found in decomposition. Data are means ± standard deviations for n = 3 replicate donors. Impacted soils that significantly differ from controls based upon Welch T-tests (p < 0.05) are presented in bold type. Asterisks indicate levels of significance: * p < 0.05, ** p < 0.01, ***p < 0.001. | | | | | | | | | |  |
| --- | --- | --- | --- | --- | --- | --- | --- | --- | --- | --- |
| **Study day** | **Location** | **Fe**  **(µg gdw^-1^)** | **Cu**  **(µg gdw^-1^)** | **Mn**  **(µg gdw^-1^)** | **Zn**  **(µg gdw^-1^)** | **Se**  **(µg gdw^-1^)** | **Co**  **(µg gdw^-1^)** | **B**  **(µg gdw^-1^)** | **Al**  **(µg gdw^-1^)** | |
| **0** | **decomposition** | 2.9 ± 0.9 | 0.04 ± 0.01 | 0.7 ± 0.4 | 0.04 ± 0.02 | 0.01 ± 0.01 | 0 ± 0 | 0.39 ± 0.16 | 9.8 ± 2 | |
|  | **control** | 2.5 ± 1.4 | 0.05 ± 0.02 | 0.5 ± 0.2 | 0.04 ± 0.01 | 0 ± 0 | 0 ± 0 | 0.46 ± 0.19 | 8.8 ± 2.5 | |
| **3** | **decomposition** | 2.7 ± 1 | 0.04 ± 0.01 | 0.6 ± 0.4 | 0.04 ± 0.02 | 0.01 ± 0.01 | 0 ± 0.01 | 0.28 ± 0.18 | 9.1 ± 3.8 | |
|  | **control** | 2.6 ± 1.5 | 0.05 ± 0.01 | 0.6 ± 0.1 | 0.04 ± 0.01 | 0.01 ± 0 | 0 ± 0 | 0.36 ± 0.13 | 8.9 ± 2.8 | |
| **5** | **decomposition** | 2.9 ± 1.2 | 0.05 ± 0.01 | 0.7 ± 0.5 | 0.04 ± 0.01 | 0.01 ± 0.01 | 0.01 ± 0.01 | 0.3 ± 0.16 | 9.5 ± 2.9 | |
|  | **control** | 2.4 ± 1.3 | 0.06 ± 0.03 | 0.6 ± 0.3 | 0.03 ± 0 | 0 ± 0.01 | 0 ± 0 | 0.36 ± 0.21 | 9.5 ± 2.8 | |
| **7** | **decomposition** | 2.9 ± 0.9 | 0.04 ± 0.01 | 0.8 ± 0.6 | 0.04 ± 0.02 | 0.01 ± 0.01 | 0.01 ± 0.01 | 0.29 ± 0.12 | 9.9 ± 3.7 | |
|  | **control** | 2.7 ± 1.6 | 0.05 ± 0.02 | 0.8 ± 0.6 | 0.04 ± 0.02 | 0.01 ± 0.01 | 0.01 ± 0.01 | 0.29 ± 0.05 | 10.8 ± 6.4 | |
| **10** | **decomposition** | 2.4 ± 1.8 | 0.04 ± 0.01 | 0.6 ± 0.4 | 0.03 ± 0.01 | 0.01 ± 0 | 0 ± 0 | 0.28 ± 0.19 | 6.7 ± 2.5 | |
|  | **control** | 2.3 ± 1.6 | 0.05 ± 0.02 | 0.5 ± 0.1 | 0.03 ± 0.01 | 0.01 ± 0.01 | 0 ± 0 | 0.33 ± 0.16 | 8.5 ± 5.8 | |
| **14** | **decomposition** | 2.1 ± 1.5 | 0.04 ± 0.01 | 0.7 ± 0.5 | 0.03 ± 0 | 0.01 ± 0.01 | 0.01 ± 0.01 | 0.32 ± 0.26 | 7.2 ± 2.7 | |
|  | **control** | 1.8 ± 1 | 0.06 ± 0.02 | 0.4 ± 0 | 0.03 ± 0.02 | 0.01 ± 0.01 | 0 ± 0 | 0.32 ± 0.1 | 6.9 ± 3.8 | |
| **17** | **decomposition** | 3.6 ± 5.4 | 0.07 ± 0.05 | **1.4 ± 0.1**** | 0.05 ± 0.03 | 0.02 ± 0.02 | 0.02 ± 0.04 | 0.56 ± 0.61 | 6.6 ± 8.9 | |
|  | **control** | 2.8 ± 1.2 | 0.05 ± 0.02 | 0.6 ± 0.2 | 0.04 ± 0.01 | 0.01 ± 0.01 | 0 ± 0 | 0.38 ± 0.12 | 9.9 ± 2 | |
| **19** | **decomposition** | 2.5 ± 3.6 | 0.06 ± 0.03 | 3.8 ± 4.3 | 0.42 ± 0.64 | 0.03 ± 0.02 | 0.01 ± 0.02 | 0.58 ± 0.58 | 4.9 ± 7 | |
|  | **control** | 3.6 ± 0.6 | 0.05 ± 0.02 | 0.8 ± 0.5 | 0.05 ± 0.01 | 0 ± 0.01 | 0.01 ± 0.01 | 0.42 ± 0.2 | 13 ± 2.3 | |
| **21** | **decomposition** | 2.5 ± 2.9 | 0.05 ± 0.02 | 4.9 ± 5.2 | **0.06 ± 0.01*** | 0.02 ± 0.03 | 0.01 ± 0 | 0.54 ± 0.61 | 4.5 ± 5.3 | |
|  | **control** | 2.5 ± 0.9 | 0.04 ± 0.02 | 0.7 ± 0.4 | 0.04 ± 0 | 0 ± 0.01 | 0 ± 0 | 0.34 ± 0.14 | 10.3 ± 1.4 | |
| **28** | **decomposition** | 0.9 ± 0.7 | 0.05 ± 0.02 | 17.1 ± 16.5 | 0.12 ± 0.04 | 0.04 ± 0.02 | 0.02 ± 0.02 | 0.89 ± 1.03 | **1.4 ± 1.1**** | |
|  | **control** | 2.7 ± 1.1 | 0.06 ± 0.03 | 0.7 ± 0.4 | 0.04 ± 0 | 0.01 ± 0.01 | 0.01 ± 0.02 | 0.43 ± 0.22 | 10.5 ± 1.9 | |
| **33** | **decomposition** | 1.9 ± 1.6 | 0.07 ± 0.02 | 28 ± 18.4 | 0.13 ± 0.07 | 0.05 ± 0.02 | 0.04 ± 0.02 | 0.92 ± 0.82 | 2.7 ± 2.8 | |
|  | **control** | 2 ± 1 | 0.05 ± 0.02 | 0.5 ± 0 | 0.04 ± 0.02 | 0.01 ± 0.01 | 0 ± 0 | 0.37 ± 0.21 | 9 ± 3.8 | |
| **35** | **decomposition** | 2.8 ± 1.5 | 0.07 ± 0.02 | 14 ± 20.5 | 0.16 ± 0.12 | 0.04 ± 0.02 | 0.03 ± 0.03 | 0.62 ± 0.39 | 4.9 ± 3.1 | |
|  | **control** | 2.6 ± 0.8 | 0.06 ± 0.03 | 0.7 ± 0.3 | 0.05 ± 0.01 | 0.01 ± 0 | 0 ± 0 | 0.39 ± 0.17 | 11.1 ± 1.9 | |
| **38** | **decomposition** | 4.2 ± 1.9 | **0.08 ± 0.01*** | 33.6 ± 33.5 | 0.18 ± 0.09 | 0.05 ± 0.03 | 0.06 ± 0.06 | 0.79 ± 0.7 | 5.3 ± 3.9 | |
|  | **control** | 2 ± 0.9 | 0.05 ± 0.01 | 0.6 ± 0.4 | 0.04 ± 0.02 | 0.01 ± 0 | 0 ± 0 | 0.35 ± 0.11 | 9.1 ± 3.5 | |
| **40** | **decomposition** | 2.1 ± 1.6 | 0.06 ± 0.03 | 25.8 ± 38.3 | 0.11 ± 0.03 | 0.04 ± 0.02 | 0.04 ± 0.04 | 0.46 ± 0.17 | 3.4 ± 2.7 | |
|  | **control** | 2 ± 0.8 | 0.05 ± 0.02 | 0.6 ± 0.6 | 0.04 ± 0.02 | 0 ± 0 | 0 ± 0 | 0.35 ± 0.12 | 8.3 ± 4.1 | |
| **42** | **decomposition** | 3 ± 1.2 | 0.07 ± 0.02 | 41.8 ± 55.2 | 0.14 ± 0.07 | 0.05 ± 0.03 | 0.1 ± 0.11 | 0.69 ± 0.54 | 2.7 ± 1.7 | |
|  | **control** | 2.1 ± 0.7 | 0.06 ± 0.02 | 0.7 ± 0.6 | 0.04 ± 0.02 | 0.01 ± 0.01 | 0 ± 0.01 | 0.4 ± 0.16 | 8.8 ± 3.8 | |
| **45** | **decomposition** | 3.6 ± 1.1 | 0.08 ± 0.02 | 30.3 ± 30.5 | **0.16 ± 0.03*** | **0.04 ± 0.01*** | 0.08 ± 0.07 | 0.69 ± 0.5 | 3.5 ± 3.2 | |
|  | **control** | 1.9 ± 0.2 | 0.06 ± 0.02 | 0.7 ± 0.5 | 0.05 ± 0 | 0.01 ± 0.01 | 0.01 ± 0 | 0.38 ± 0.13 | 8.4 ± 3 | |
| **47** | **decomposition** | 5.3 ± 3.5 | 0.06 ± 0 | 35 ± 43.7 | 0.13 ± 0.07 | 0.04 ± 0.03 | 0.07 ± 0.09 | 0.62 ± 0.42 | 4.1 ± 4.2 | |
|  | **control** | 1 ± 0.4 | 0.05 ± 0.02 | 0.6 ± 0.3 | 0.03 ± 0 | 0.01 ± 0.01 | 0 ± 0 | 0.35 ± 0.13 | 6.5 ± 2.6 | |
| **49** | **decomposition** | 4.9 ± 3.9 | 0.08 ± 0.01 | 62.3 ± 43.2 | **0.22 ± 0.07*** | 0.06 ± 0.03 | 0.16 ± 0.13 | 0.68 ± 0.46 | 3.5 ± 2.8 | |
|  | **control** | 1.9 ± 0.6 | 0.06 ± 0.01 | 0.7 ± 0.6 | 0.05 ± 0.01 | 0 ± 0 | 0 ± 0.01 | 0.38 ± 0.13 | 8.9 ± 3.4 | |
| **54** | **decomposition** | 4.1 ± 2.4 | 0.08 ± 0.02 | 21.2 ± 13.9 | **0.07 ± 0.01*** | 0.03 ± 0.02 | 0.07 ± 0.05 | 0.59 ± 0.41 | 5.6 ± 3.1 | |
|  | **control** | 2.8 ± 0.7 | 0.05 ± 0.02 | 0.8 ± 0.5 | 0.04 ± 0 | 0.01 ± 0.01 | 0 ± 0.01 | 0.37 ± 0.14 | 11.6 ± 2.3 | |
| **56** | **decomposition** | **7 ± 1.1**** | 0.12 ± 0.04 | 27.8 ± 38.4 | 0.18 ± 0.06 | 0.04 ± 0.03 | 0.13 ± 0.14 | 0.48 ± 0.25 | 14.4 ± 6.1 | |
|  | **control** | 2.5 ± 0.7 | 0.05 ± 0.01 | 0.7 ± 0.4 | 0.04 ± 0 | 0.01 ± 0.01 | 0 ± 0 | 0.34 ± 0.12 | 11.1 ± 2.5 | |
| **61** | **decomposition** | **8.2 ± 2.1*** | 0.1 ± 0.02 | 23.8 ± 13.3 | **0.14 ± 0.04*** | **0.04 ± 0.01*** | 0.11 ± 0.07 | 0.56 ± 0.47 | 13.7 ± 10 | |
|  | **control** | 3.2 ± 1 | 0.05 ± 0.03 | 0.8 ± 0.6 | 0.04 ± 0.01 | 0.01 ± 0 | 0.01 ± 0.01 | 0.36 ± 0.13 | 11.6 ± 2 | |
| **66** | **decomposition** | 3 ± 1.6 | 0.08 ± 0.04 | 20.4 ± 14.1 | 0.11 ± 0.04 | **0.04 ± 0.01*** | 0.06 ± 0.03 | 0.55 ± 0.41 | 5.7 ± 4.5 | |
|  | **control** | 3 ± 0.6 | 0.05 ± 0.02 | 0.9 ± 0.6 | 0.05 ± 0.01 | 0 ± 0 | 0 ± 0.01 | 0.33 ± 0.12 | 13 ± 3.4 | |
| **75** | **decomposition** | 5 ± 6.2 | 0.08 ± 0.05 | 39.9 ± 53.6 | 0.15 ± 0.11 | 0.04 ± 0.03 | 0.09 ± 0.09 | 0.33 ± 0.1 | 11.1 ± 16.7 | |
|  | **control** | 2.8 ± 1.1 | 0.04 ± 0 | 0.8 ± 0.4 | 0.04 ± 0.01 | 0.01 ± 0.01 | 0 ± 0 | 0.27 ± 0.09 | 11.1 ± 0.3 | |
| **89** | **decomposition** | **11.2 ± 1.6**** | 0.11 ± 0.04 | **27.7 ± 3.3**** | 0.15 ± 0.06 | **0.04 ± 0.01**** | **0.12 ± 0.04*** | 0.62 ± 0.45 | 25.4 ± 10.4 | |
|  | **control** | 1.2 ± 0.5 | 0.05 ± 0.02 | 0.5 ± 0.3 | 0.03 ± 0.01 | 0.01 ± 0.01 | 0 ± 0 | 0.33 ± 0.09 | 6.2 ± 2 | |
| **103** | **decomposition** | **27.3 ± 4.2**** | 0.13 ± 0.08 | 13.7 ± 6.3 | 0.43 ± 0.36 | 0.02 ± 0.01 | 0.1 ± 0.09 | 0.47 ± 0.25 | **58.9 ± 15.8*** | |
|  | **control** | 1.2 ± 0.4 | 0.05 ± 0.01 | 0.4 ± 0.1 | 0.02 ± 0.01 | 0 ± 0.02 | 0 ± 0 | 0.36 ± 0.16 | 5.7 ± 2.6 | |
| **117** | **decomposition** | 25.3 ± 16.9 | **0.19 ± 0.03**** | 20.1 ± 13.8 | 0.49 ± 0.22 | **0.03 ± 0***** | 0.54 ± 0.42 | 0.41 ± 0.19 | 39.3 ± 26.8 | |
|  | **control** | 0.8 ± 0.2 | 0.04 ± 0.01 | 0.3 ± 0.1 | 0.01 ± 0.01 | 0 ± 0 | 0 ± 0 | 0.34 ± 0.1 | 4.2 ± 0.9 | |
| **122** | **decomposition** | 24.3 ± 20 | 0.16 ± 0.07 | 15.6 ± 14.2 | 0.22 ± 0.12 | 0.02 ± 0.01 | 0.24 ± 0.32 | 0.43 ± 0.12 | 40.9 ± 15.4 | |
|  | **control** | 0.9 ± 0.3 | 0.04 ± 0 | 0.4 ± 0.2 | 0.02 ± 0 | 0 ± 0.01 | 0 ± 0 | 0.32 ± 0.08 | 4.7 ± 1.2 | |
