# Supplementary material for: Soil elemental changes during human decomposition
Source: PLoS One. 2023 Jun 13;18(6):e0287094. doi: 10.1371/journal.pone.0287094 (PMC10263346; doi:10.1371/journal.pone.0287094)
Supplement: Attachment — Submitted filename: PONE_D_23_02805 Response to reviewers.docx [file pone.0287094.s007.docx]

**PONE-D-23-02805 Response to Reviewers**

Reviewer comments are in italics; our responses are in **bold blue**. Line numbers are in reference to the marked-up track changes document, and are cross-referenced to original line numbers.

**Reviewer 1:**

*The control sites were 2m from each body. What was the rationale for this specific distance? Based on the results it seems to be sufficient but no justification is provided in the text.*

**Thank you for pointing out this omission. The following text has been added in order to clarify the choice of control spacing: “The selection of control sites 2 m was to ensure they were not impacted by human decomposition products; this was based on previous research demonstrating limited (< 1 m) lateral transport of decomposition products through soil [6, 10, 24].” (lines 150-152)**

*Table 2. Please clarify what ‘Trial 3’ in the table title refers to.*

**We appreciate your catching this error. The text “Trial 3” has been removed. (line 154)**

*Figure 3. Branching in the cluster analysis appears to indicate that Mn and Se cluster with Group 1 rather than Group 2. Please outline why these elements are included in Group 1.*

**The reviewer makes a good point. In the hierarchical cluster analysis (Fig 3), they cluster distantly with group 1. However in our PCA model, they cluster strongly with group 1. We opted to use the PCA model as our main guidance in grouping the elements. To clarify this decision, we have added the following text to the discussion:**

**“In contrast with elemental grouping levels derived from PCA ordination, hierarchical clustering based upon correlative relationships suggests an alternative view that Mg, Ca, Mn, Se, and B are related to group 1 elements rather than forming their own unique grouping level. Correlative relationships are mathematically strong with respect to elemental concentration changes and their timings, however caution should be exercised in interpretation to avoid conflating apparent patterns derived from a combination of tissue decomposition, pH, and the soil exchange. For example, unlike Ca^2+^ and Mg ^2+^, which are highly correlated and behave similarly in soil, Mn and Se appear to derive from differing sources (soil and soft tissue, respectively) and by dissimilar mechanisms, thus suggesting that their correlation is largely mathematical rather than mechanistic. It is our view that these elements (Mg, Ca, Mn, Se, and B) can be classified, albeit loosely, into their own separate grouping level.” (lines 577-587).**

**Reviewer 2:**

*Comments to the line 236: The change in mobility under the influence of soil acidity applies to Al, but also to the other trace metals. For example, the availability and mobility of Cu and Zn will increase in low pH environments due to the chemical for in which these metals are present in the soil solutions. Maybe it’s enough to add this trace metal to the list with the rest, then?*

**This is a good suggestion. We have added the following to clarify our intent: “The mobility of Al (as well as Fe, Cu, Mn, Zn, and Co) can alter under soil acidification frequently observed during human decomposition…” (lines 237-238)**

*Line 401: Szelecz et al. (2018)…observed a decrease in soil pH (in effect, acidification) under the influence of decomposing Sus scofa carcasses. A study by Fancher et al. (2017)…did not conclusively demonstrate that decomposition of human cadavers decreases soil pH. The results were divergent. Barton et al. (2020)…in a study compared the decomposition of human and pig cadavers. The effect of cadaver decomposition, regardless of species, affected changes in soil pH levels in a similar way. In my opinion, it is difficult to determine a clear trend here.*

**The reviewer has correctly pointed out that changes in soil pH are not as clear when considering longer decomposition timelines, however, trends in pH are more clear during early soft tissue decomposition, which is what we are referencing here. In general, surface human decomposition tends to result in soil acidification during early soft tissue decomposition, whereas the same time frame in animal decomposition tends to result in alkalinization; these correspond with the period of time associated with our study (initial placement through active and advanced decay, and greatest tissue mass loss). Szelecz et al. (2018) supports this: Figure 3 shows an increase in pH for pig treatments prior to day 84 during active and early advanced decay. Fancher et al. (2017) and Barton et al. (2020) were both chronosequences, not longitudinal studies, so we exercise caution in deriving pH trajectories from these. We acknowledge that there is some variability across studies, so to that end have altered the language to make it less definitive. This line now reads: “…less often during animal decomposition.” (line 403)**

**We think there is increasingly compelling evidence that humans and animals exhibit differing decomposition rates and chemistries, so we have kept this differentiation in our discussion. Early pH changes differ in quite a pronounced manner between humans and animals. In lines 69-73 we state the following: “While frequently used as human surrogates, there are differences between pig and human tissue composition [31], decomposition patterns [8, 32, 33], and soil chemistry responses, particularly with respect to pH [8, 24] (but see [5, 8-11, 14, 16-20]).” Given that temporal changes to pH are a controlling factor in elemental mobilization in soil, and there is evidence suggesting that this might be species-dependent, we think it’s important to keep this nuance.**

*Line 446: In study of Cholewa et al (2018)…after about 200 days, the level of inorganic form of phosphorus (P2O5) in the soil with buried pig limbs differed from the control fields, as well as* *from each other (the depth of buried animal tissues was a differentiating factor).*

**We would like to thank the reviewer for the suggestion of comparing our elemental analysis results with Cholewa et al. (2022). There are several key differences between the experimental design of Cholewa et al. (2022) and that of our study that preclude direct comparison between them, e.g.: Cholewa et al. (2022) discusses results derived from the decomposition of pig tissue remains buried in soil plots composed of well-aerated (sieved) garden soil; our study which describes the surface decomposition of whole human remains placed upon an in-situ soil profile. Thus we have focused on making our comparisons to other surface-decomposition studies of whole human remains to avoid conflating 1) surface and burial environments, and 2) whole and partial remains. Decomposition rates and chemistries in both scenarios differ markedly and Cholewa et al. (2022) refers to these differences many times in their study when discussing limitations of their interpretation. While we have included a very select few references from the burial and tissue decomposition literature we have attempted to carefully use them as supporting references only after surface decomposition of whole remains have been utilized, and not in a capacity that they are the sole source of corroborative argument. In order to ensure clarity with respect to our focus we have modified the wording of line 79 to state: “…impacted by surface decomposition of whole human remains…”**

*Line 477: Results of Cholewa et al (2018)…confirm that it was the same with potassium. Plants growing at the site where the pigs’ limbs were buried did not have higher levels of K, although there was more of it in the soil – perhaps the plants are not responsible for its loss (as well as for the loss of Na, as demonstrated by the authors of the reviewed paper).*

**Please see previous comment regarding this study.**

*Line 517: Was the drop in Ca levels related to the appearance of adipocere? Fiedler et al. (2004) suggested that the calcium was bound in adipocere. Fiedler, Schneckenberger, & Graw, Characterization of Soils Containing Adipocere. Arch Environ. Contam Toxicol 47, 561-568 (2004).*

**This is a very interesting idea, and we appreciate the reviewer suggesting this data interpretation, as we had not considered it! We have now added the following text to the discussion: “Interestingly, soil solution concentrations of both Ca and Mg were reduced beginning on day 56 during the period in which active decay was transitioning to advanced decay. At this time the CDI was still “wet” and exhibiting evidence of fat decomposition products. Shortly thereafter (day 80) CDIs were observed to have dried and formed hard crusts in which visible adipocere was present. The formation of adipocere includes interactions between fatty acids, Na, and K, leading to the formation of fatty acid salts, and there is evidence that Ca and Mg present in the soil can displace Na and K, thus forming Ca and Mg salts [55, 56]. Given that our extraction methodology was water-based and thus would not solubilize adipocere, it is entirely possible that we have observed dual phenomena: the evolution of Ca and Mg from the soil exchange complex and into the soil solution, followed by the incorporation of both cations into adipocere. This possibility is supported by Lühe et al. [30], in which both Ca and Mg fatty acid salt concentrations increased over time following human decomposition. Further study would be needed to quantify fatty acid and fatty acid salt content of these soils to directly link elemental changes with fat decomposition transformation products, and to begin the creation of an elemental soil budget.” (lines 544-558)**
